# Genomic Characterization of Potential Opportunistic Zoonotic *Streptococcus parasuis* Isolated in China

**DOI:** 10.3390/pathogens14040395

**Published:** 2025-04-18

**Authors:** Gang Liu, Yu Liu, Zhikang Jiang, Kang Liu, Xianwen Wang, Juyuan Hao, He Kong, Yajie Yu, Zicheng Ding, Min Li, Xianjie Han

**Affiliations:** College of Veterinary Medicine, Qingdao Agricultural University, Qingdao 266109, China

**Keywords:** *Streptococcus parasuis*, whole-genome sequencing, virulence gene, antibiotic resistance gene, mobile genetic elements

## Abstract

(1) Background: *S. parasuis* is a potential opportunistic zoonotic pathogen that can infect pigs, cattle, and humans, composed of former members of *S. suis* serotypes 20, 22, and 26. In recent years, unclassified serotypes and a serotype 11 *S. parasuis* have been discovered. (2) Methods: We characterized two *S. parasuis* strains (FZ1 and FZ2) isolated from brain samples of paralyzed pigs and examined evolutionary divergence among 22 available *S. parasuis* and 8 serotype 2 *S. suis* genomes through whole-genome sequencing and comparative genomic analysis. We compared virulence genes (VGs) and antibiotic resistance genes (ARGs) and analyzed mobile genetic elements (MGEs) in FZ1 and FZ2. (3) Results: Comparative genomics revealed that *srtC*, *ctpV*, and *sugC* may represent key virulence determinants in *S. parasuis*, although their pathogenic potential appears attenuated compared to serotype 2 *S. suis*. In addition, *S. parasuis* exhibited primary resistance to aminoglycosides, macrolides, tetracyclines, and oxazolidinones, while demonstrating heightened susceptibility to oxazolidinone-class antibiotics. Moreover, we found an important association between MGEs and antibiotic resistance in *S. parasuis* FZ1 and FZ2. (4) Conclusions: This study provides new insights into the genomic and evolutionary characteristics of *S. parasuis* and provides a new basis for the study of bacterial pathogenesis and drug resistance in the future.

## 1. Introduction

*Streptococcus suis* (*S. suis*) is an important zoonotic pathogen that can be transmitted to humans via direct contact with infected pigs or consumption of undercooked contaminated pork products, constituting a potential risk to public health and safety [1]. *S. suis* is Gram-positive and can be divided into 35 serotypes according to different capsular polysaccharide antigens [1]. Serotype 2 *S. suis* is the most prevalent serotype with zoonotic potential, especially in Europe and Asia [2]. In 2015, *S. suis* serotypes 20, 22, and 26 were reclassified as *Streptococcus parasuis* (*S. parasuis*) on the basis of average nucleotide identity, 16S ribosomal RNA (rRNA), and biochemical characteristics [3]. In contrast to *S. suis*, clinical reports of *S. parasuis* remain limited, primarily due to the absence of reliable diagnostic methods capable of differentiating these species [4,5]. In 2018, a novel polymerase chain reaction (PCR) method specifically targeting *S. parasuis* was developed on the basis of nucleotide sequence comparisons of *recN* with *S. parasuis* and its close relatives [4].

The presence of *S. parasuis* in diseased pigs and calves with pneumonia or systemic infection (meningitis, arthritis, endocarditis, or septicemia) suggesting its pathogenic potential in livestock [6,7,8]. *S. parasuis* is widespread in swine populations globally, with only sporadic reports of infection in calves, and given the lack of clinical isolates, the importance of *S. parasuis* for public health is underestimated [3]. Recently, three human *S. parasuis* infection cases with pneumonia and arthritis have been reported in China, indicating that the zoonotic pathogen *S. parasuis* is an emerging threat to public health [3,9]. At present, the presence of *S. parasuis* has recently been reported in a few countries, such as China, Japan, Canada, and Switzerland [10], but the genome characteristics and pathogenesis of *S. parasuis* still need further study. In this study, two new strains of *S. parasuis* were isolated from the brains of two paralyzed pigs, the genomic characteristics of *S. parasuis* isolates FZ1 and FZ2 were analyzed, and evolutionary divergence was assessed through whole-genome sequencing and comparative genomic analysis of 22 *S. parasuis* genomes and 8 serotype 2 *S. suis* genomes. This study provides new insights into the genomic and evolutionary characteristics of *S. parasuis* and provides a new basis for the study of bacterial pathogenesis and drug resistance in the future.

## 2. Materials and Methods

### 2.1. Bacterial Isolation and Identification

In May 2023, brain samples were aseptically collected from two paralytic pigs at a pig farm in Shandong Province and promptly transported to the laboratory under refrigeration. Deep tissue samples were aseptically excised using sterile scissors and then incubated on blood agar plates at 37 °C for 24 h under aerobic conditions. Single colonies were selected and purified using blood agar, and the morphological characteristics of the isolated bacteria were determined by Gram staining and biochemical identification with the API 20 strep *Streptococcus* Biochemical Identification Kit (Biomerieux, Marcy I’Etoile, France). Two suspected strains of *S. parasuis* were named FZ1 and FZ2 and stored at −80 °C in broth containing 15% glycerol for further analysis.

### 2.2. Phylogenetic Analysis

Strains were grown aerobically in 25 mL of brain heart infusion broth (Haibo Biotechnology, Qingdao, China) at 37 °C with shaking at 180 rpm. For preliminary identification, a small fragment of the 16S rRNA gene was amplified using universal primer set (27F 5′-AGAGTTTGATCCTGGCTCAG-3′; 1492R 5′-GGTTACCTTGTTACGACTT-3′) [11]. The PCR mixture consisted of 8.5 μL of nuclease-free water, 12.5 μL of 2 × Taq Master Mix, 1 μL of each primer, and 2 μL of genomic DNA in a total volume of 25 μL. The PCR conditions were initial denaturation for 3 min at 95 °C, followed by 30 cycles of amplification for 15 s at 95 °C, 15 s at 55 °C, 90 s at 72 °C, and a final elongation at 72 °C for 5 min (GeneAmp PCR System 2700; Applied Biosystems, Foster City, CA, USA). PCR products were visualized by 1% (*w*/*v*) agarose gel electrophoresis in 1 × TAE buffer and then sequenced at Sangon Biotech Co., Ltd. (Shanghai, China). Spliced sequences were compared with online data in the NCBI database (http://www.ncbi.nlm.nih.gov, accessed on 5 April 2024), and multiple sequence alignments were carried out using the ClustalW program in MEGA 7.0 [12]. A phylogenetic tree was constructed by applying the neighbor-joining (NJ) method with the genetic distances calculated by the Kimura 2-parameter model. Bootstrap analysis was also performed in a total of 1000 replicates for the NJ analysis.

### 2.3. DNA Extraction

The genomic DNA was extracted following the instructions of the TIANamp Bacteria DNA Kit (TIANGEN BIOTECH, Beijing, China). Qualified genomic DNA was used as the starting material for sequencing and library construction. The quality and integrity of genomic DNA was assessed using 1% agarose gel electrophoresis and densitometry compared to the appropriate size standards. Meanwhile, DNA yield and purity were measured using NanoDrop™ 2000 spectrophotometer (Thermo Fisher Scientific, Waltham, MA, USA) and TBS-380 fluorometer (Turner BioSystems Inc., Sunnyvale, CA, USA). High-quality DNA was used to conduct further research.

### 2.4. Genome Sequencing, Assembly, and Annotation

In this study, a combination of the Illumina NovaSeq 6000 and PacBio Sequel II platforms was used to complete the genome maps of the two *S. parasuis* at Beijing Novogene Bioinformatics Technology Co., Ltd. (Beijing, China). An Illumina NovaSeq library and a PacBio library were constructed. Draft genome sequencing was carried out using the Illumina NovoSeq platform (Illumina, San Diego, CA, USA). Approximately 1 µg DNA was sheared to construct a sequencing library of 400~500 bp insertion fragments. Paired-end 2 × 150 bp reads were then sequenced. After quality control, the Illumina sequencing data were preliminarily assembled using SPAdes v3.15.3 [13]. For PacBio sequencing, SMRTbell library inserts (20 kb) were sequenced, and subreads shorter than 500 bp were removed. The PacBio sequences were error-corrected, binned, and then assembled through the Canu v2.2 assembler [14], and Pilon v1.24 [15] was used for assembly polishing with Illumina short reads to improve genome quality. To determine the presence of any plasmids, the filtered Illumina reads were mapped through SOAPdenovo2 [16] to the bacterial plasmid database. Circos v0.69-6 [17] was used to construct the circular map of the genome and comprehensively display the relevant information. The rRNA genes were identified through RNAmmer v1.2 [18], and the tRNA genes were identified through tRNAscan-SE v2.0.8 [19] with default settings. The above assembled sequences were used to predict coding genes through Glimmer3 v3.02. The predicted gene sequences were translated and searched against the National Center for Biotechnology Information (NCBI) nonredundant database, the Gene Ontology (GO) database, the protein families (Pfam) database, the Clusters of Orthologous Groups (COG) database, and the Kyoto Encyclopedia of Genes and Genomes (KEGG) database for annotation.

### 2.5. Comparative Genomic Analysis

To measure the similarity among the strains, the digital DNA-DNA hybridization (dDDH) and the Orthologous Average Nucleotide Identity (OrthoANI) were calculated between pairs of genomes. The dDDH was calculated with GGDC (Genome-to-Genome Distance Calculator 3.0 https://ggdc.dsmz.de/ggdc.php#, accessed on 3 May 2024). OrthoANI was calculated through ChunLab’s Orthologous Average Nucleotide Identity Tool (OAT), with an algorithm demarcation cutoff of 95~96% [20]. The genome sequences of FZ1 and FZ2 were submitted to PubMLST (https://pubmlst.org/, accessed on 10 May 2024) [21] and compared with the *S. suis* database.

Different online tools have been used to search for genetic transfer. The VFanalyzer tool of VFDB web-services (http://www.mgc.ac.cn/VFs/, accessed on 15 May 2024) [22] was used to estimate VGs. Amino acid sequences of VGs were compared with multiple-aligned sequences. ResFinder web-services (Center for Genomic Epidemiology (dtu.dk), accessed on 22 May 2024) [23] was used to detect ARGs and kept default settings. To search for CRISPR-Cas sequences, the genomes were analyzed through the CRISPRCasFinder (https://crisprcas.i2bc.paris-saclay.fr/, accessed on 23 May 2024) [24] online tool. PHASTEST (https://phastest.ca/, accessed on 25 May 2024) [25] was utilized for identifying prophage sequences; ICEfinder (https://tool2-mml.sjtu.edu.cn/ICEberg3/ICEfinder.php, accessed on 26 May 2024) [26] was used to detect ICEs. The identification of genomic islands was performed with IslandViewer 4 (https://www.pathogenomics.sfu.ca/islandviewer/, accessed on 26 May 2024) [27] through the IslandPath-DIMOB method.

In addition, all 22 sets of acquirable genome data for *S. parasuis* were downloaded from the National Centre for Biotechnology Information (NCBI) on June 2024, and pangenomic analysis of *S. parasuis* genomes including FZ1 and FZ2 was performed through BPGA v1.3 [28]. The raw sequencing dataset of *S. parasuis* SUT-319 was assembled through SPAdes v3.15.5 [13]. The VGs and ARGs of *S. parasuis* were compared with those of 8 serotype 2 *S. suis* strains that were randomly selected through R 4.3.2. A progressive Mauve (v2.4.0) [29] algorithm was introduced to observe the sequence identities of FZ1, FZ2, and *S. suis* S735. All *S. parasuis* and *S. suis* genomes used in this study are shown in Table 1.

### 2.6. Mice Survival Test

An experimental infection model in mice was designed to assess the pathogenicity of *S. parasuis* strains FZ1 and FZ2 by comparing the survival rates of infected mice. A total of 50 female C57BL/6 mice (6 weeks old) were randomly allocated into two infection groups (2 replicates per group, 10 mice per replicate) and one control group (2 replicates, 5 mice per replicate). C57BL/6 mice were intraperitoneally injected with 5 × 10^7^ CFUs of test strains in 1 mL of THB or THB alone (control). Mortality was recorded every 6 h for the first 24 h and every 12 h thereafter 72 h post-infection. Survival curves were generated using the Kaplan–Meier method, and experiments were performed in duplicate [9].

## 3. Results

### 3.1. Bacterial Characteristics and Phylogenetic Analysis of S. parasuis FZ1 and FZ2

The two isolated strains were demonstrated to be Gram-positive bacteria, and the colonies on blood agar were milky white, translucent, circular, and nonpigmented with α-hemolytic bacteria. Biochemical assays revealed that both strains tested negative for the pyruvate (VP) and hippurate (HIP) tests. Additionally, no enzymatic activity was detected for β-glucosidase (ESC), pyrrolidonyl arylamidase (PYRA), α-galactosidase (α GAL), β-glucuronidase (β GUR), β-galactosidase (β GAL), alkaline phosphatase (PAL), arginine dehydrolase (ADH), or leucine arylamidase (LAP). Acid was produced from trehalose (TRE), starch (AMD), and glycogen (GLYG), but not from ribose (RIB), L-arabinose (ARA), mannitol (MAN), sorbitol (SOR), lactose (LAC), inulin (INU), or raffinose (RAF).

To identify the pathogenic species accurately, a 1517 bp 16S rRNA sequence of the isolated strains was amplified and sequenced. BLASTN analysis confirmed that the sequences belonged to the genus *Streptococcus*. The 16S rRNA genes of strains FZ1 and FZ2 presented the greatest identification (>99%) with those of *S. parasuis*, with the highest sequence similarity with *S. parasuis* SUT-447 (99.93% and 99.86%, respectively), followed by those with *S. parasuis* SUT-7 (99.45% and 99.52%, respectively) and *S. parasuis* SUT-319 (99.43% and 99.50%). In contrast, the 16S rRNA of the isolates exhibited 96–97% identity with *S. suis* and 95–96% nucleotide identity with *S. ruminantium*.

**Figure 1:**
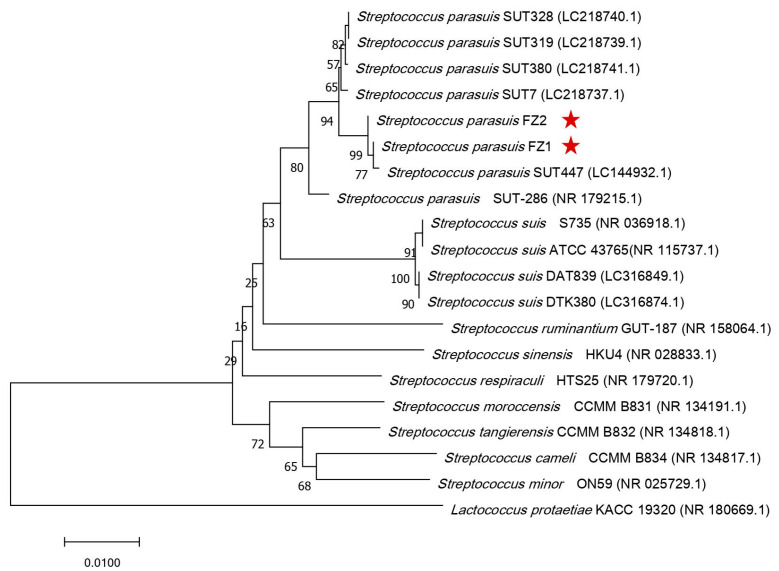
Phylogenetic tree based on 16S rRNA gene sequences of *S. parasuis* FZ1, FZ2, and closely related species. The phylogenetic tree was created through the neighbor-joining method. The 16S rRNA sequence of *Lactococcus protaetiae* KACC 19320 was used as an outgroup to root the trees. Bootstrap values (1000 replicates) are shown at the branch points. The scale bar indicates 0.01 nucleotide substitutions per nucleotide position. The red stars mark the locations of strains FZ1 and FZ2.

To further verify the nucleotide BLAST results, a detailed phylogenetic tree was constructed, which revealed the relationships of *S. parasuis* FZ1 and FZ2 with closely related *Streptococcus* species. Moreover, the phylogenetic tree revealed that FZ1, FZ2, and *S. parasuis* SUT-447 clustered together in the same clade, demonstrating their high genetic relatedness. Distinct lineages were formed with *S. parasuis* SUT-7, *S. parasuis* SUT-380, *S. parasuis* SUT-328, *S. parasuis* SUT-319, and *S. parasuis* SUT-286 (Figure 1). Therefore, the phylogenetic analysis confirmed that the two isolated strains were novel strains of *S. parasuis*; thus, we designated them *S. parasuis* FZ1 and FZ2.

### 3.2. Genomic Features and Gene Functional Analysis of S. parasuis Isolates

The genomes of strains FZ1 and FZ2 consisted of 2,054,729 bp and 2,032,338 bp circular chromosomes with mean G+C contents of 39.46% and 39.44%, respectively. The complete genome sequences of FZ1 and FZ2 contained 2085 and 2125 predicted coding sequences (CDSs), respectively, while both contained 58 tRNAs, 12 rRNAs, 4 5S rRNAs, 4 16S rRNAs, and 4 23S rRNAs. In addition, three plasmids—pFZ2-1 (16,488 bp), pFZ2-2 (6065 bp), and pFZ2-3 (5488 bp) were identified in FZ2 (Figure S1).

The gene functions were predicted through GO, COG, and KEGG analyses. The COG-annotated genes of FZ1 and FZ2 were both divided into 23 COG subclasses, with 1697 and 1691 COG-annotated genes, respectively. The most enriched categories were “translation, ribosome structure, and biogenesis” (222 and 221 genes, respectively), followed by “amino acid transport and metabolism” (179 and 175 genes, respectively), “transcription” (140 and 132 genes, respectively), “replication, recombination, and repair” (110 and 121 genes, respectively), “carbohydrate transport and metabolism” (105 and 100 genes, respectively), and “cell wall/membrane/envelope biogenesis” (103 and 116 genes, respectively) (Figure S2).

GO analysis revealed that 560 and 551 protein-encoding genes in FZ1 and FZ2, respectively, were categorized into biological process, cellular component, and molecular function categories. The most annotated biological process function was translation (54 and 54 genes, respectively). The integral components of the membrane (119 and 109 genes, respectively) and cytoplasm (83 and 87 genes, respectively) were the top two enriched cellular components. For molecular functions, DNA binding (86 and 82 genes, respectively) and ATP binding (83 and 72 genes, respectively) were the most abundant (Figure S3).

KEGG analysis identified 1255 and 1228 annotated genes in FZ1 and FZ2, respectively, distributed across six categories. Among them, the most populated class was represented by metabolism pathways (929 and 926 genes, respectively), followed by genetic information processing (156 and 155 genes, respectively), environmental information processing (165 and 161 genes, respectively), human diseases (77 and 86 genes, respectively), cellular processes (71 and 68 genes, respectively), and organismal systems (28 and 28 genes, respectively). The most abundant KEGG pathways were the global and overview maps (365 and 368 genes, respectively) within the metabolism category, followed by amino acid metabolism (114 genes, entirely) and membrane transport (111 and 113 genes, respectively) (Figure S4).

### 3.3. Comparative Genomic Analysis of S. parasuis FZ1 and S. parasuis FZ2

**Figure 2:**
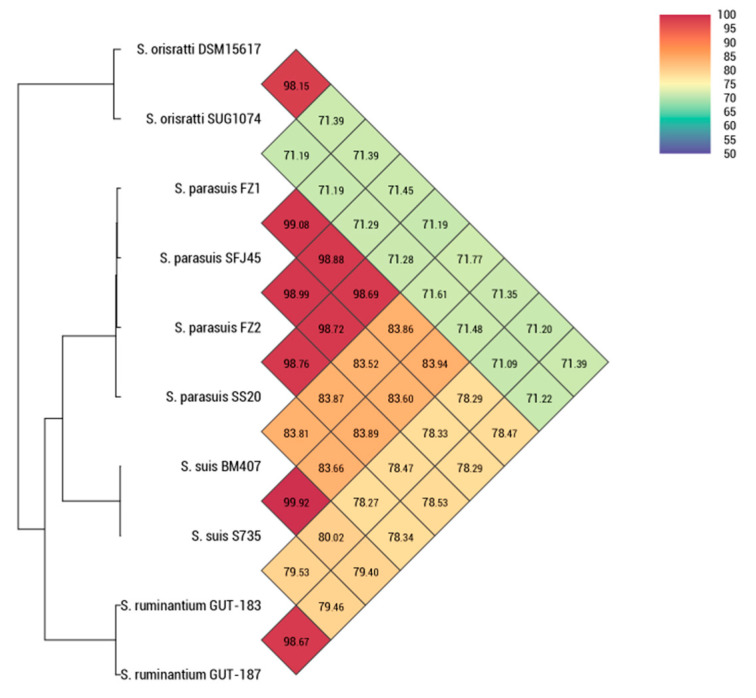
Values of OrthoANI for the strains of interest.

The dDDH and OrthoANI values of the isolated strains *S. parasuis* FZ1 and FZ2 were compared with those of the *S. parasuis*, *S. suis*, *S. orisratti*, and *S. ruminantium* strains. The dDDH values of *S. parasuis* FZ1 and FZ2 compared with those of *S. parasuis* SFJ45 (92.10% and 90.70%, respectively), and *S. parasuis* SS20 (87.8% and 88.8%, respectively) exceeded the 70% cutoff points, whereas those of *S. suis* BM407 (28.6% and 28.30%, respectively), *S. suis* S735 (28.60% and 28.30%, respectively), *S. ruminantium* GUT-183 (23.60% and 23.40%, respectively), *S. ruminantium* GUT-187 (23.10% and 23.00%, respectively), *S. orisratti* SUG1074 (25.00% and 24.70%, respectively), and *S. orisratti* DSM15617 (27.20% and 26.70%, respectively) were all below the 70% cutoff points recommended for delineating species. Similarly, the OrthoANI values for FZ1 and FZ2 compared to *S. parasuis* strains exceeded the 95–96% cutoff, while those for *S. suis*, *S. orisratti*, and *S. ruminantium* were below this threshold, which indicated that the isolated strains *S. parasuis* FZ1 and FZ2 were *S. parasuis* (Figure 2*)*.

*S. parasuis* were compared with the pubMLST database of *S. suis.* Seven allele sequences of *aroA*, *cpn60*, *dpr*, *gki, mutS*, *recA*, and *thrA* were found in both FZ1 and FZ2. Submission of allele profiles of seven housekeeping genes to the PubMLST database confirmed that FZ1 and FZ2 belong to two different sequence types; hence, following verification, two new sequence types (STs) were assigned to the respective allele combinations ST2909 and ST2910 (Table 2).

One type of CRISPR-Cas system (Type IC) was found in *S. parasuis* FZ1 but was absent in *S. parasuis* FZ2. The chromosome of FZ1 carried one phage, one ICE, and nine GIs, of which the ICE and two GIs carried AMR genes, while FZ2 carried three phages and eights GIs, and only one phage and one GI carried AMR genes (Table 3). These factors, along with others yet to be investigated, collectively determine the emergence and transfer of AMR between *S. parasuis* and different bacterial strains.

Pangenomic analysis was performed on FZ1, FZ2, and 22 available *S. parasuis* strains, which had a total of 28,368 core genes (1182 genes per genome), 15,794 accessory genes (average of 658.08 genes per genome), 1680 unique genes (average of 70 genes per genome), and 141 exclusively absent genes (average of 5.88 genes per genome). To investigate the phylogenetic relationships among these 24 *S. parasuis* isolates, a neighbor-joining tree was constructed on the basis of core genome alignment (Figure S5). The phylogenetic tree revealed distinct clustering patterns based on the isolation sources of the strains. *S. parasuis* isolated from humans (BS26, BS27, NN1, 7500, 221006) and cows (86-5192, 10-36905) formed two different clades, whereas *S. parasuis* isolated from pigs (SUT-7, 88-1861, SUT-328, SUT-319, SUT-380, SS-5819, FZ2, FZ1, SFJ45, H35, SS20, SS17, AH0906, SUT-286, 89-4109-1, and SUT-503) formed multiple clades (Figure S5). Moreover, *S. parasuis* from China (FZ1, FZ2, SFJ45, H35, SS17, and SS20) formed a large clade, with the exception of *S. parasuis* AH0906, whereas *S. parasuis* from other countries formed multiple clades (Figure S5). These findings indicate that the phylogenomic analysis of the core genome may reveal differences between strains according to their isolated sources and regions.

The proteins encoded by all the genes of *S. parasuis* were annotated in the Database of Clusters of Orthologous Genes (COGs). Only assigned COG functional genes were considered. The different functional preferences of the core, accessory, and unique were analyzed. Core genes were predominantly associated with COG categories J (translation, ribosomal structure, and biogenesis), E (amino acid transport and metabolism), and R (general function prediction only), which were present in a greater proportion compared to accessory and unique genes. In contrast, accessory genes of *S. parasuis* were more often associated with the COG categories R, K (transcription), L (replication, recombination, and repair), and G, and unique genes were more often associated with the COG categories M (cell wall/membrane/envelope biogenesis), K, L, G, and R (Figure S5). This finding indicates that the core genes of *S. parasuis* are preferred for different physiological and biological functions, and that the functions of accessory and unique genes are involved in genetic evolution, contribute to species diversity and provide selective advantages.

**Figure 3:**
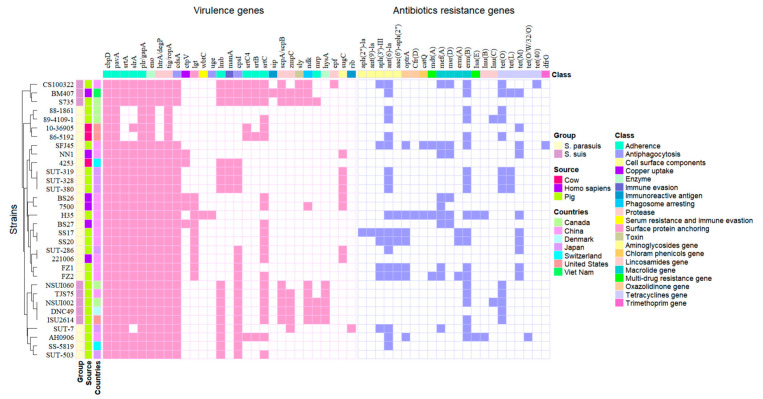
Heatmap of virulence genes and antibiotic resistance genes of 24 *S. parasuis* and 8 serotype 2 *S. suis* strains.

The 135 virulence genes of *Streptococcus* from VFDB were investigated in 24 *S. parasuis* and 8 *S. suis* strains, of which 29 virulence genes were present in these strains and were further analyzed (Figure 3). The analysis revealed that virulence traits related to adherence, enzymes, and proteases were detected consistently in all of the examined strains, such as adherence associated virulence genes *cbpD* (100.00%, 32/32), *pavA* (100.00%, 32/32), *plr/gapA* (100.00%, 32/32), the enzyme associated gene *eno* (100.00%, 32/32), and the protease gene *tig/ropA* (100%, 32/32). In addition, seven other virulence genes are associated with adherence, such as *slrA* (87.50%, 28/32), *srtA* (87.50%, 28/32), *lmb* (50%, 16/32), *srtC4* (18.75%, 6/32), *srtB* (15.63%, 5/32), *srtC* (65.63%, 21/32), and *mrp* (12.50%, 4/32); three virulence genes are associated with antiphagocytosis, including *cdsA* (87.50%, 28/32), *cpsI* (62.5%, 20/32), and *uge* (3.13%, 1/32); *hysA* (15.63%, 5/32) is associated with enzymes; four genes, including *htrA/degP* (87.50%, 28/32), *scpA/scpB* (25.00%, 8/32), *zmpC* (21.88%, 7/32), and *epf* (3.13%, 1/32), are associated with proteases; and the other eight categories of virulence genes, including *lgt* (31.25%, 10/32), *ndk* (28.13%, 9/32), *sugC* (25.00%, 8/32), *ctp V*(15.63%, 5/32), *manA* (21.88%, 7/32), *sly* (9.38%, 3/32), *wbtC* (3.13%, 1/32), *sip* (3.13%, 1/32), and *rib* (3.13%, 1/32), were also identified.

Among the 29 virulence genes in *S. parasuis*, 75.86% (22/29) of the virulence genes were present in pig *S. parasuis*, 51.72% (15/29) present in human *S. parasuis* and 55.17% (16/29) present in cow *S. parasuis*. *S. parasuis* from pigs in Japan (58.62%, 17/29) and China (58.62%, 17/29) had the highest detection percentages, followed by *S. parasuis* from Switzerland (37.93%, 11/29) and Canada (20.69%, 6/29) from pigs. The percentages of *lmb* (100%, 8/8), *cpsI* (100%, 8/8), *srtC* (100%, 8/8), *scpA/scpB* (100%, 8/8), and *ndk* (100%, 8/8) detected in 8 *S. suis* type 2 strains were greater than those detected in 24 *S. parasuis* strains (33.33%, 50%, 54.17%, 0.00%, and 4.17%, respectively).

We found that the *wbtC* and *uge* genes of *S. parasuis* H35 were unique genes; the *scpA/scpB*, *mrp*, and *hysA* genes of SUT-319 were unique genes; and the *zmpC* and *rib* genes of SUT-7 were unique genes in *S. parasuis*. Moreover, the *sip*, *scpA/scpB*, *epf*, *sly*, *mrp*, and *hysA* genes are specific to *S. suis*, and the *ctpV*, *lgt*, *wbtC*, *uge*, *sugC*, and *rib* genes are specific to *S. parasuis* and exist only in human *S. parasuis*. These findings indicate that there are differences in the virulence genes of *S. parasuis* strains isolated from different sources and regions and that there are differences between *S. parasuis* and serotype 2 *S. suis* strains (Figure 3). In addition, compared with *S. parasuis* BS26, *S. parasuis* FZ1 lacks the *srtC*, *ctpV*, and *sugC* genes, whereas *S. parasuis* FZ2 lacks the *ctpV* and *sugC* genes. Combined with the results of the mouse survival test, we speculate that the *srtC*, *ctpV*, and *sugC* genes are important virulence factors for the pathogenicity of *S. parasuis*.

A total of 21 and 10 ARGs divided into eight categories were detected from *S. parasuis* and *S. suis*, respectively. The analysis revealed that ARGs related to aminoglycosides, macrolides, and tetracycline were detected consistently in *S. parasuis* and serotype 2 *S. suis*. The aminoglycoside gene *ant(6)-Ia* (62.50%, 15/24) and macrolide gene *erm(B)* (54.17%, 13/24) were the two genes with the highest detection percentages in *S. parasuis*, followed by macrolide *mef(A)* (37.50%, 9/24), tetracycline *tet(M)* (37.50%, 9/24), *tet(O)* (25.00%, 6/24), aminoglycoside *aph(3′)-III* (25.00%, 6/24), and oxazolidinone *optrA* (29.17%, 7/24). The aminoglycosides ARGs *aph(2″)-Ia* (4.17%), *ant(9)-Ia* (4.17%), and *aac(6′)-aph(2″)* (20.83%); the oxazolidinone ARG *cfr(D)* (4.17%); the chloram phenicols ARG *cat(Q)* (8.33%); the multidrug ARGs *mdt(A)* (12.50%) and *Isa(E)* (8.33%); the macrolide ARGs *msr(D)* (20.83%) and *erm(A)* (12.50%); the lincosamide ARGs *lnu(B)* (8.33%) and *lnu(C)* (4.17%); the tetracycline ARGs *tet(L)* (8.33%) and *tet(O/W/32/O)* (4.17%); and the trimethoprim ARG *dfrG* (4.17%) were also identified in 24 *S. parasuis*. In addition, the carriage of ARGs varies among different countries and source isolates. With respect to the isolated regions, Chinese *S. parasuis* from pigs had the richest resistance categories (8/8) of ARGs, followed by *S. parasuis* from pigs in Canada (4/8) and Japan (3/8). With respect to the isolated sources, *S. parasuis* from pigs contained the highest number of ARGs (21/21), followed by *S. parasuis* from cows (4/21) and humans (3/21). Moreover, *erm(B)* (75.00%, 6/8) and *tet(O)* (87.50%, 7/8) had the highest detection percentages in the studied strains of serotype 2 *S. suis*, followed by *ant(6)-Ia* (25.00%), *aph(3′)-III* (12.50%), *mef(A)* (12.50%), *msr(D)* (12.50%), *tet(L)* (12.50%), and *tet(40)* (12.50%), while the remaining ARGs were not detected in serotype 2 *S. suis*. These findings indicated that the antibiotic resistance of *S. parasuis* and *S. suis* was similar.

**Figure 4:**
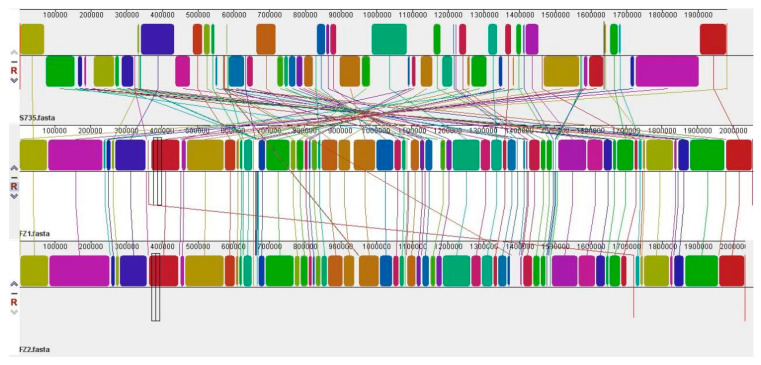
Collinearity between *S. parasuis* FZ1, FZ2, and serotype 2 *S. suis* strain S735. Each contiguously colored region is a locally collinear block. LCBs below a genome’s center line are in the reverse complement orientation relative to the reference genome. Lines between genomes trace each orthologous LCB through every genome. Areas that are completely white were not aligned and probably contain sequence elements specific to a particular genome.

Collinearity revealed that the genomes of FZ1 and FZ2 presented obvious fragmented deletions, inversions, and translocations compared with those of *S. suis* S735, whereas the collinear set of matched colored regions of FZ1 and FZ2 presented significant similarities (Figure 4). These results suggest that although the two types of strains belong to *Streptococcus*, there are significant differences in their evolutionary histories. This may be related to their long-term independent evolution in different microenvironments. More in-depth analyses of the completely sequenced genomes of *S. parasuis* strains and investigations of the specific functions of important genes are necessary in the future.

### 3.4. Differential Survival Rates in Mice Infected with S. parasuis FZ1 and S. parasuis FZ2

**Figure 5:**
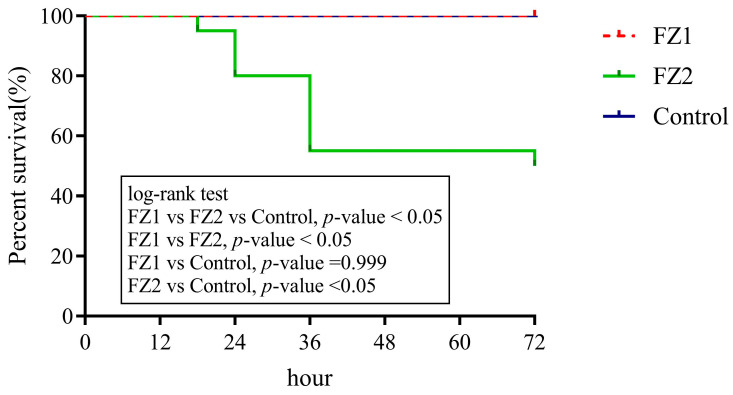
Survival curves of mice infected with 5 × 10^7^ CFUs of *S. parasuis* strains FZ1, FZ2, or THB only as the control group. The survival rates of different groups were compared through the Log-rank test.

Mice infected with *S. parasuis* FZ1 exhibited significantly higher survival rates compared to those challenged with FZ2 (*p* < 0.05). The survival rate of the mice infected with 5 × 10^7^ CFUs of *S. parasuis* strain FZ1 was 100% at 24 h post-infection, whereas that of the mice infected with strain FZ2 was 80% at the same time point. The survival rates of the mice infected with *S. parasuis* FZ1 and FZ2 were 100% and 50%, respectively, at 72 h post-infection (Figure 5).

## 4. Discussion

*S. parasuis* has been isolated from pigs, humans, and cows, and has characteristics similar to those of *S. suis*; however, its enzymatic activity and acid production differ [5]. *S. parasuis* is often isolated from healthy pigs, which has led to the notion that *S. parasuis* may be included in the normal microbiota of pigs. *S. parasuis* can be concomitantly isolated from diseased pigs and the bacterium itself has a low degree of virulence [4]. Exploring the genomic characteristics at the whole-genome level or comparing some phenotypic determinants could improve our understanding of the molecular and evolutionary changes in bacteria [9,30]. In this study, the genomes of *S. parasuis* isolates FZ1 and FZ2 were analyzed and compared with those of acquirable *S. parasuis* and its closely related serotype 2 *S. suis* genomes obtained from a public database.

To evaluate the potential virulence of *S. parasuis* strains FZ1 and FZ2, survival curves of FZ1 and FZ2 were generated, and virulence genes were compared with all available genomes of *S. parasuis* from public databases. Deficiency of the *srtC* gene significantly affects the synthesis of pili structure, leading to a significant reduction in bacterial adhesion, invasion, and virulence [31]. Mice infected with the Δ*ctpV* strain (*Mycobacterium tuberculosis*) exhibit a reduced immune response to bacteria and a significantly increased lifetime [32]. The lifetime of mice infected with the Δ*sugC* mutant strain (*Mycobacterium*) through the aerosol pathway was significantly longer than that of those infected with the wild-type strain [33]. Compared with those in *S. parasuis* BS26, the *ctpV* and *sugC* genes in *S. parasuis* FZ1 and *S. parasuis* FZ2 were absent, and the *srtC* gene was absent in FZ1. In addition, significant differences in survival were detected between FZ1 and FZ2-infected groups and the BS26-infected group. Therefore, the *srtC*, *ctpV*, and *sugC* genes were crucial for the pathogenicity of *S. parasuis*, and their role in *S. parasuis* could be evaluated in further research.

The massive quantities of bacterial genomic data generated have facilitated in-depth analyses of bacteria for pangenomic studies [34]. Different numbers of accessory and unique genes were present in 24 *S. parasuis*. These dispensable genes and strain-specific genes are categorized as secondary genes, delineating the partially shared and strain-specific attributes of a species. These characteristics distinguish strains from one another and contribute to species diversity. Partially shared and strain-specific genes play roles that are not essential for growth but provide selective advantages, such as adaptation to different hosts and antibiotic resistance, indicating that owing to the presence of accessory genes and specific genes, *S. parasuis* is diverse. Structural variation in bacterial genomes is an important evolutionary driver. Genomic rearrangements, such as inversions, duplications, and insertions, can regulate gene expression and promote niche adaptation [35].

In addition, the distribution of virulence and antibiotic-resistant genes of *S. parasuis* in different strains may be correlated with the virulence of *S. parasuis* and the isolation source and regions of the strains. Genomic analysis revealed that *S. parasuis* and serotype 2 *S. suis* frequently harbor resistance genes for aminoglycosides, oxazolidinones, macrolides, and tetracyclines, indicating that antibiotic resistance is similar between *S. parasuis* and serotype 2 *S. suis*. Although oxazolidinones have not been approved for veterinary use, the potential for co-selection and co-transfer of oxazolidinone resistance genes may be facilitated by the widespread use of other antimicrobial agents, such as phenicols, in animal populations [36].

Various common ARGs and some high-risk ARGs (i.e., *bla*_ampC_, *bla*_OXA-1_, and *bla*_TEM-1_) were prevalent in family livestock waste, and the pollution of tetracycline resistance genes was the most serious in these family livestock farms [37,38]. Infections caused by antibiotic-resistant bacteria are a major threat to global public health [39]. Mobile genetic elements (MGEs) play a key role in the intra- and interspecies horizontal transfer of antimicrobial resistance determinants. For example, several MGEs carrying ARG determinants for tetracyclines, macrolides, aminoglycosides, and chloramphenicol have been identified in *S. suis* [40]. Previously, a *tet(M)*- and *aadE*-carrying pathogenicity island (PAI) was identified in China, which was found to be unusual in streptococcal toxic shock syndrome (STSS)-causing *S. suis* strains from pigs. The *tet(W)*-carrying prophage ΦSsuD.1 was found in a *S. suis* strain from humans, and chimeric and tandem ICEs in streptococci have been reported [40]. In this study, different MGEs carrying ARGs were predicted in *S. parasuis* FZ1 and FZ2. Our findings indicate that there may be potential genetic exchange between the two strains and other bacterial strains, and that the interaction of MGEs in FZ1 and FZ2 may increase MGE diversity and complexity.

The collinearity analysis revealed structural variation in the evolutionary process of *S. parasuis* and serotype 2 *S. suis*. This may be related to their long-term independent evolution in different microenvironments. These analyses provide a basis for comparative genomics research and the study of genome-wide evolutionary dynamics.

## 5. Conclusions

In summary, this study sequenced the whole genomes of *S. parasuis* FZ1 and FZ2 strains in China and examined all the genomes of *S. parasuis* from public databases to explore their similarities and differences from those of its close relative serotype 2 *S. suis*. We clearly revealed that the *srtC*, *ctpV*, and *sugC* genes may be important for the pathogenicity of *S. parasuis*. There may be differences in virulence among different *S. parasuis* strains, which may be related to the isolation source and regions of the strains. In addition, *S. parasuis* is mainly resistant to aminoglycosides, macrolides, tetracyclines, and oxazolidinones, which is similar to serotype 2 *S. suis*, and there is an important association between MGEs and antibiotic resistance in *S. parasuis* FZ1 and FZ2. In addition, long-term independent evolution in different microenvironments may have led to structural variations between *S. parauis* and serotype 2 *S. suis* and differences in the virulence genes of the two types of *Streptococcus*. This study elucidates molecular mechanisms underlying antibiotic resistance and pathogenic virulence in *S. parasuis*, establishing a framework for future investigations into bacterial pathogenesis and antimicrobial resistance dynamics, and providing a better understanding of the evolution of *S. suis*.

## Figures and Tables

**Table 1:** Information on all *S. parasuis* and *S. suis* genomes used.

Species	Strains	Source	Location	Year	Accession No.	Complete?	Length
*Streptococcus parasuis*	SUT-380	Pig	Japan	2013	AP024277.1	Y	2,109,881
*Streptococcus parasuis*	SUT-503	Pig	Japan	2014	AP024280.1	Y	2,065,066
*Streptococcus parasuis*	SUT-286	Pig	Japan	2013	AP024276.1	Y	2,197,342
*Streptococcus parasuis*	SUT-7	Pig	Japan	2012	AP024275.1	Y	2,202,836
*Streptococcus parasuis*	BS27	Homo sapiens	China	2018	JAETXU000000000.1	N	1,909,795
*Streptococcus parasuis*	BS26	Homo sapiens	China	2018	CP069079.1	Y	1,932,292
*Streptococcus parasuis*	H35	Pig	China	2018	CP076721.1	Y	2,186,318
*Streptococcus parasuis*	4253	Cow	Switzerland	2018	SHGT00000000.1	N	1,881,656
*Streptococcus parasuis*	86–5192	Cow	United States	1980	ALLG00000000.1	N	2,110,166
*Streptococcus parasuis*	88–1861	Pig	Canada	1980	ALLW00000000.1	N	2,272,254
*Streptococcus parasuis*	89–4109-1	Pig	Canada	1980	ALLL00000000.1	N	2,176,728
*Streptococcus parasuis*	SUT-319	Pig	Japan	/	DRX016753	N	2,129,893
*Streptococcus parasuis*	SUT-328	Pig	Japan	/	BOJH00000000.1	N	2,126,590
*Streptococcus parasuis*	10–36,905	Cow	United States	2010	WNXH00000000.1	N	2,148,541
*Streptococcus parasuis*	SFJ45	Pig	China	2017	CP102747.1	Y	2,015,398
*Streptococcus parasuis*	7500	Homo sapiens	China	2022	CP128410.1	Y	2,008,266
*Streptococcus parasuis*	221006	Homo sapiens	China	2022	CP137602.1	Y	1,985,497
*Streptococcus parasuis*	SS17	Pig	China	2021	CP090522.1	Y	1,984,594
*Streptococcus parasuis*	NN1	Homo sapiens	China	2020	CP073632.1	Y	1,971,006
*Streptococcus parasuis*	SS20	Pig	China	2021	CP086728.1	Y	1,961,908
*Streptococcus parasuis*	AH0906	Pig	China	2009	JANFLX000000000.1	N	2,203,067
*Streptococcus parasuis*	SS-5819	Pig	Switzerland	2022	JAVIID010000001.1	N	2,130,485
*Streptococcus parasuis*	FZ1	Pig	China	2021	CP170759	Y	2,054,729
*Streptococcus parasuis*	FZ2	Pig	China	2022	CP170760	Y	2,032,338
*Streptococcus suis*	BM407	Homo sapiens	Viet Nam	2004	FM252032.1	Y	2,146,229
*Streptococcus suis*	S735	Pig	Canada	/	CP003736.1	Y	1,980,887
*Streptococcus suis*	TJS75	Pig	China	2015	CP095162.1	Y	2,368,195
*Streptococcus suis*	NSUI060	Pig	Canada	2008	CP012911.1	Y	2,285,232
*Streptococcus suis*	ISU2614	Pig	United States	2014	CP031377.1	Y	2,163,384
*Streptococcus suis*	CS100322	Pig	China	2010	CP024050.1	Y	2,137,649
*Streptococcus suis*	DNC49	Pig	Denmark	/	CP102140.1	Y	2,137,486
*Streptococcus suis*	NSUI002	Pig	Canada	2008	CP011419.1	Y	2,255,345

**Table 2:** MLST of *S. parasuis* FZ1 and FZ2.

Strains Name	Multi-Locus Allelic Profile							ST	Genome Accession
	***aroA***	***cpn60***	***dpr***	***gki***	***mutS***	***recA***	***thrA***		
FZ1	299	440	56	83	83	67	155	2909	CP170759
FZ2	299	83	172	635	83	67	228	2910	CP170760

**Table 3:** Summary of genetic traits present in the *S. parasuis* FZ1 and *S. parasuis* FZ2 strains.

	FZ1	FZ2	Does Prophages/ICEs/GIs Contain Resistance Genes?	
			FZ1	FZ2
CRISPR-Cas	Type IC	/	/	/
Prophages	SpsFZ1-P1	SpsFZ2-P1	N	N
		SpsFZ2-P2		Y (*aac(6′)-aph(2″)*, *ant(6)-Ia*, *mdt(A)*, *erm(B)*)
		SpsFZ2-P3		N
ICEs	SpsFZ1-ICE1	/	Y (*tet(M)*)	/
GIs	SpsFZ1-GI1	SpsFZ2-GI1	N	N
	SpsFZ1-GI2	SpsFZ2-GI2	Y (*ermC*, *aacA-aphD*, *OptrA,* *ANT(9)*, *bacA*, *aadK*)	Y (*ermC*, *aacA-aphD*, *OptrA*, *ANT(9)*, *aadK*)
	SpsFZ1-GI3	SpsFZ2-GI3	Y (*ermC*, *mef*, *aadK*, *aph3-III*)	N
	SpsFZ1-GI4	SpsFZ2-GI4	N	N
	SpsFZ1-GI5	SpsFZ2-GI5	N	N
	SpsFZ1-GI6	SpsFZ2-GI6	N	N
	SpsFZ1-GI7	SpsFZ2-GI7	N	N
	SpsFZ1-GI8	SpsFZ2-GI8	N	N
	SpsFZ1-GI9	/	N	/

## Data Availability

The data presented in the study are deposited in the NCBI repository, accession number PRJNA1165511.

## Supplementary Materials

The following supporting information can be downloaded at: https://www.mdpi.com/article/10.3390/pathogens14040395/s1, Figure S1: (A,B) show the structure and organization of the genomes of *S. parasuis* FZ1 and *S. parasuis* FZ2, respectively. The circles (from the outside to the inside) indicate the predicated scale in bp, coding sequences, rRNA and tRNA, GC content, and GC-skew. The coding sequences with different COG annotation functions are shown in different colors; Figure S2: (A,B) are COG function annotations of *S. parasuis* FZ1 and FZ2, respectively; Figure S3: (A,B) are GO function annotations of *S. parasuis* FZ1 and *S. parasuis* FZ2, respectively; Figure S4: (A,B) are KEGG function annotations of *S. parasuis* FZ1 and *S. parasuis* FZ2, respectively; Figure S5: Pangenomic analysis of 24 *S. parasuis*. (A) Phylogenomic tree based on the neighbor-joining method. The red stars mark the locations of strains FZ1and FZ2. (B) Cluster of orthologous groups (COGs) functional classification of core genes and accessory genes of *S. parasuis*.
