# Supplementary material for: Genomic Characterization of Potential Opportunistic Zoonotic Streptococcus parasuis Isolated in China
Source: Pathogens. 2025 Apr 18;14(4):395. doi: 10.3390/pathogens14040395 (PMC12030105; doi:10.3390/pathogens14040395)
Supplement: Supplementary file 1 [file pathogens-14-00395-s001.zip › pathogens-3539660-supplementary.pdf]

Supplementary Materials

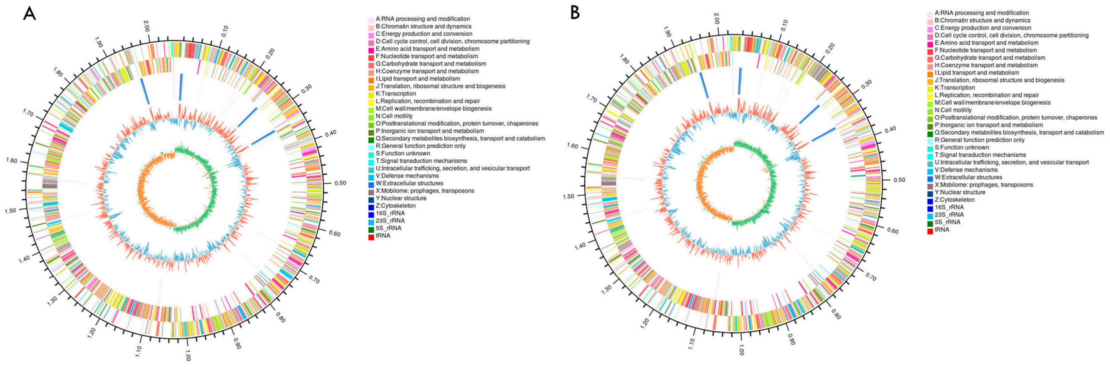

Figure S1. A and B show the structure and organization of the genomes of *S. parasuis* FZ1 and *S. parasuis* FZ2, respectively. The circles (from the outside to the inside) indicate the predicated scale in bp, coding sequences, rRNA and tRNA, GC content, and GC-skew. The coding sequences with different COG annotation functions are shown in different colors.

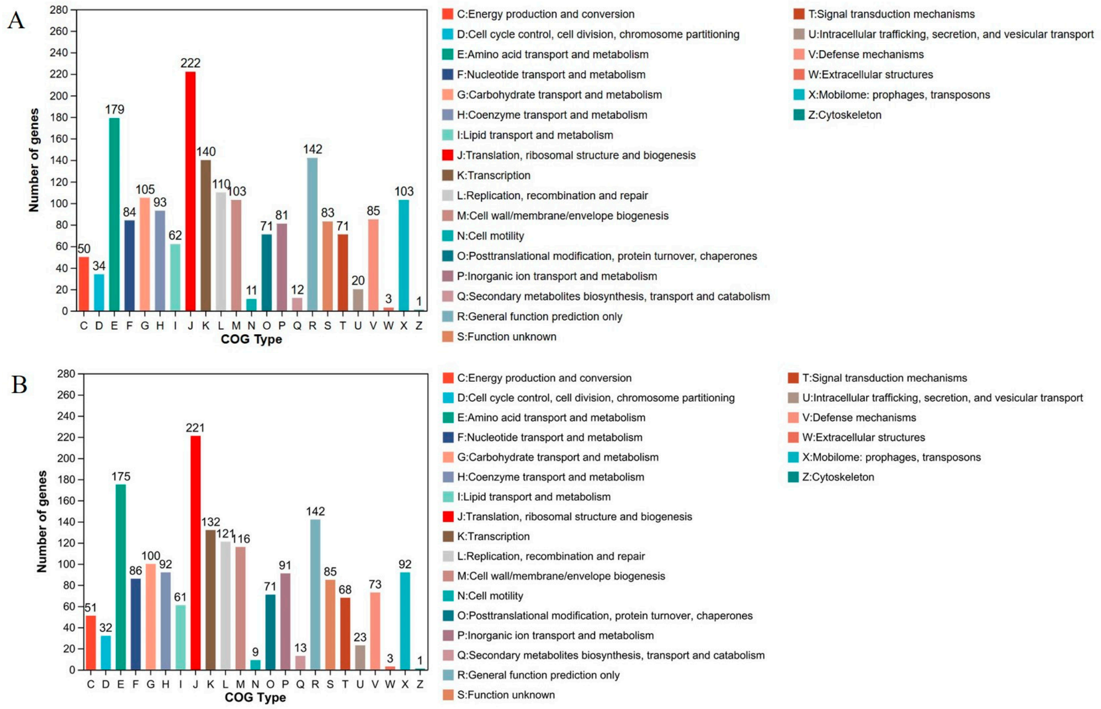

Figure S2. A and B are COG function annotations of *S. parasuis* FZ1 and FZ2, respectively.

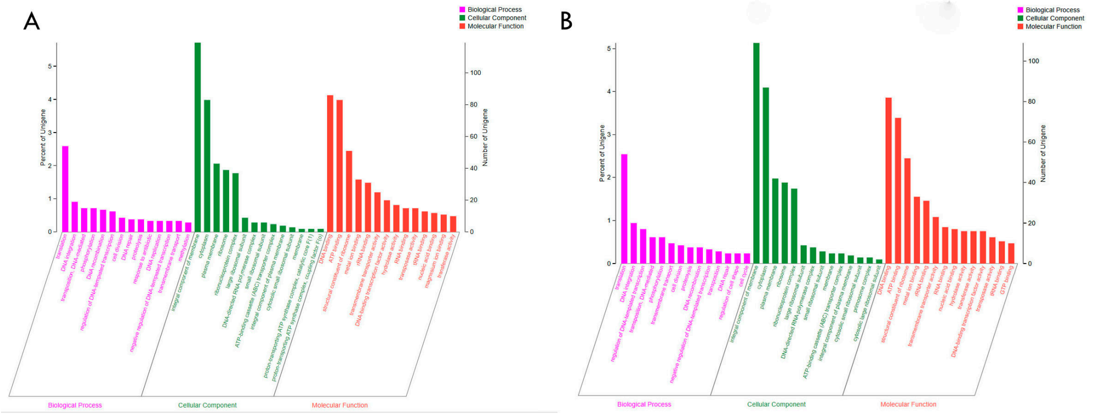

Figure S3. A and B are GO function annotations of *S. parasuis* FZ1 and *S. parasuis* FZ2, respectively.

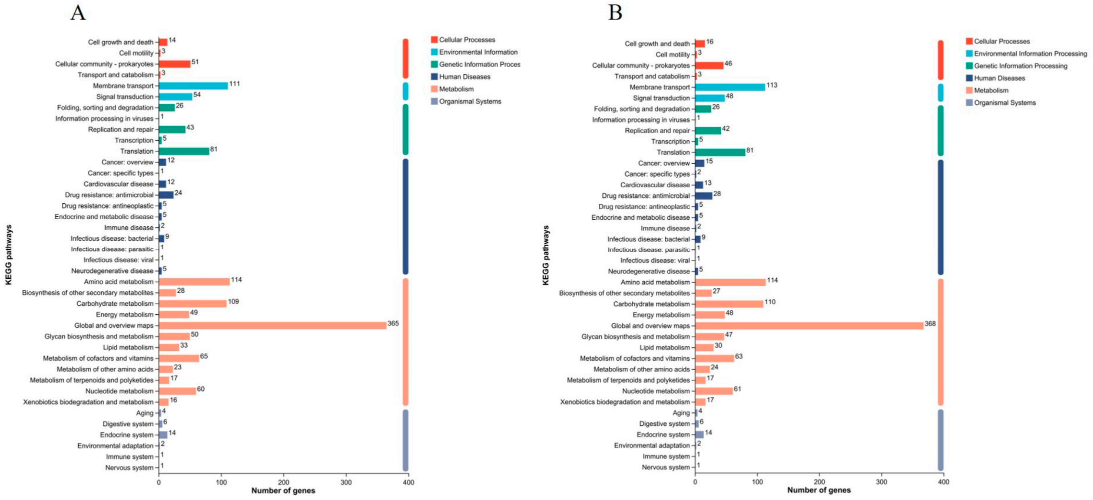

Figure S4. A, B are KEGG function annotations of *S. parasuis* FZ1 and *S. parasuis* FZ2, respectively.

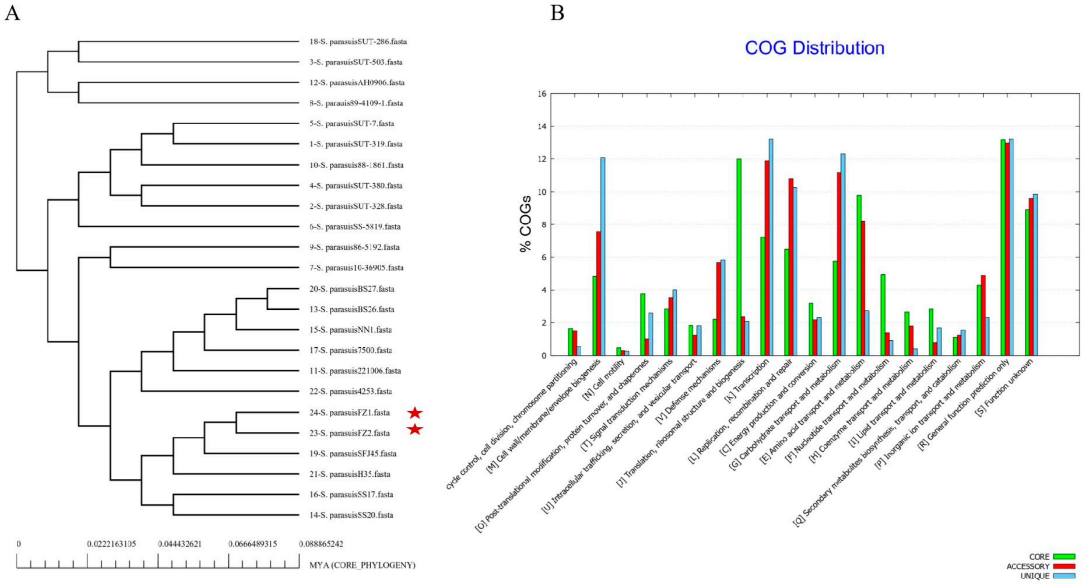

Figure S5. Pan genomic analysis of 24 *S. parasuis*. **A** Phylogenomic tree based on the neighbor-joining method. The red stars mark the locations of strains FZ1and FZ2. **B** Cluster of orthologous groups (COGs) functional classification of core genes and accessory genes of *S. parasuis*.
